# Association between MnSOD Activity and Cognitive Impairment in Unmedicated First-Episode Schizophrenia: Regulated by *MnSOD* Ala-9Val Gene Polymorphism

**DOI:** 10.3390/antiox11101981

**Published:** 2022-10-04

**Authors:** Dong Mei Wang, Rong Rong Zhu, Yang Tian, Kadir Uludag, Jia Jing Chen, Hui Xia Zhou, Li Wang, Thomas R. Kosten, Xiang Yang Zhang

**Affiliations:** 1 CAS Key Laboratory of Mental Health, Institute of Psychology, Chinese Academy of Sciences, Beijing 100101, China; 2 Department of Psychology, University of Chinese Academy of Sciences, Beijing 100049, China; 3 Department of Psychiatry and Behavioral Sciences, Baylor College of Medicine, Houston, TX 77030, USA

**Keywords:** schizophrenia, MnSOD, cognitive function, Ala-9Val polymorphism

## Abstract

The imbalance between pro-oxidants and antioxidants is thought to be responsible for aging and cognitive impairment in many degenerative diseases, including schizophrenia (SZ). As the first antioxidant enzyme to detoxify superoxide radicals in mitochondria, manganese superoxide dismutase (MnSOD) activity and its functional polymorphism of Ala-9Val have been found to be associated with SZ. In this study, we explored the association between MnSOD activity, *MnSOD* Ala-9Val polymorphism and cognitive dysfunction in unmedicated first-episode (UMFE) SZ patients, which has not been examined. We recruited 234 UMFE SZ patients and 232 healthy controls (HC) and evaluated them with Repeated Battery for the Assessment of Neuropsychological Status (RBANS), plasma MnSOD activity and *MnSOD* Ala-9Val (rs4880) polymorphism. In addition, we used the Positive and Negative Syndrome Scale (PANSS) to assess the severity of patients’ psychopathological symptoms. Compared with HC, UMFE patients showed extensive cognitive impairment on RBANS, and had higher MnSOD activity. *MnSOD* Ala-9Val polymorphism was not associated with SZ susceptibility and cognitive impairment, but only affected MnSOD activity in patients. Moreover, only in SZ patients with Val homozygotes, MnSOD activity was significantly correlated with cognitive impairment, especially in RBANS total score, visuospatial/constructional and attention index scores. Our results suggest that cognitive impairment is associated with MnSOD activity in patients with first-episode SZ, which may be regulated by *MnSOD* Ala-9Val polymorphism.

## 1. Introduction

Cognitive impairment is a pervasive feature of schizophrenia (SZ). It occurs before the onset of psychosis and is independent of the clinical symptoms of SZ [1]. More than 80% of SZ individuals exhibit deficits in at least one cognitive domain, manifested as overall changes in attention, language ability, memory, executive function and social function [2,3]. Cognitive impairment has an important functional impact on the daily life of patients, and previous studies have shown that cognitive impairment is the best predictor of long-term functional outcomes [3,4]. Current antipsychotics are effective in relieving psychotic symptoms and reducing the risk of relapse, but they cannot treat cognitive impairment of SZ patients [5]. To a certain extent, this is due to a lack of understanding of the underlying pathological mechanisms associated with cognitive impairment in SZ patients.

Oxidative stress (OS) is a general oxidation-related imbalance that is known to result in cell dysfunction or cell death [6]. Neurons in the brain are particularly sensitive to OS because of their high lipid content and high metabolic rate [7]. Several studies have shown that OS is related with the severity of symptoms in SZ patients [8,9]. The antioxidant system can neutralize reactive oxygen species (ROS) by supplying reductive equivalents (electrons) to ROS and reverse oxidative damage in the brain [10]. As the first antioxidant enzyme in the antioxidant defense system, superoxide dismutase (SOD) promotes superoxide anion radicals (O^2−^) to produce hydrogen peroxide (H_2_O_2_), which is subsequently detoxified by catalase to oxygen and water [11]. SOD includes three different metal cofactor isomers in the human brain: copper (Cu) and zinc (Zn) isomers exist in the cytoplasm and extracellular space, while manganese isomer (MnSOD) is located in the mitochondrial substrate. Due to its location, MnSOD is an important enzyme that controls ROS formation and plays an important role in numerous biochemical and molecular processes essential for the survival of aerobic life [12,13,14]. It has been reported that the activity of MnSOD in peripheral blood is disrupted in chronic [15,16] and first-episode SZ patients [17]. Another important study using the postmortem brain tissue of SZ patients found that the level of MnSOD was elevated in the prefrontal cortex [18]. The human *MnSOD* gene is located on chromosome 6 and has been identified as a linkage candidate in genome-wide linkage studies in SZ patients [19,20]. The Ala-9Vla polymorphism (rs4880) is the most widely investigated functional polymorphism of the *MnSOD* coding gene. The substitution from T to C (GTT to GCT), that is, from valine to alanine, causes the structure of the mitochondrial targeting domain to change from β-sheet to α-helix, thereby increasing the formation of active *MnSOD* tetramer in mitochondria [21,22]. Previous studies on the contribution of *MnSOD* polymorphism to SZ susceptibility are inconsistent [23,24,25,26,27]. The inclusion of subjects of different races in different studies may explain the inconsistent results. A recent meta-analysis study has shown that the Ala-9Val genotype is not associated with SZ susceptibility, or tardive dyskinesia, which is a common motor syndrome in SZ patients [15].

Excessive production of ROS in mitochondria can lead to DNA mutations and protein/lipid oxidation, which in turn leads to mitochondrial dysfunction [28], being considered to be a crucial potential cause of cognitive decline in brain aging and several diseases (i.e., Alzheimer’s disease (AD) and Parkinson) [29,30,31]. Superoxide anions are a member of ROS and SOD protects cells from the deleterious effects of superoxide anions in the organism [13]. There are many studies regarding the relationship between SOD and cognitive performance. For example, a recent study shows that SOD is an independent factor leading to cognitive deficits in patients with cerebral small vessel disease [32]. In an animal study, it was found that overexpression of SOD prevented AD-related learning and memory deficits, and reduced hippocampus superoxide radicals [33]. In addition, antioxidant treatment can also reverse the working-learning deficits and hippocampal long-term potentiation (LTP) and significantly increase the activities of SOD and another antioxidant enzyme in the hippocampus and cerebral cortex in AD animal model [34]. However, studies on the link between SOD and cognitive impairment in SZ individuals are inconsistent [16,35,36,37,38]. Our previous studies have shown that SOD activity, especially MnSOD activity, is closely related to cognitive performance of patients with chronic SZ [16,23,37], but these results are not repeated in other chronic [35,39] or first-episode [36] SZ patients. The history of antipsychotic drugs, patient status (chronic or acute episode), sample size and genetic polymorphisms may be the main reasons for these inconsistencies [40,41].

In this study, patients with unmedicated first-episode (UMFE) SZ were included to maximize control of confounding factors, including disease duration, the effect of antipsychotic drugs and related psychiatric and medical comorbidities [42]. Therefore, we studied the association between cognitive performance and MnSOD activity of individuals with UMFE SZ, and further examined whether the *MnSOD* Ala-9Val polymorphism modulated this relationship. We hypothesized that the *MnSOD* Ala-9Val polymorphism may affect the activity of MnSOD, which may lead to cognitive impairment in SZ patients. The purposes of this study were: (1) to analyze whether *MnSOD* Ala-9Val variant regulates MnSOD activity in SZ patients and healthy controls (HC); (2) to explore the association between MnSOD activity and cognitive performance in SZ patients and HC; (3) to detect the effect of *MnSOD* Ala-9Val polymorphism on cognitive performance of these two groups; and (4) to investigate whether the *MnSOD* Ala-9Val genotype would regulate the association between MnSOD activity and cognitive function.

## 2. Material and Methods

### 2.1. Subjects

Our sample included 234 patients who were hospitalized in Beijing Hui-Long-Guan Hospital and followed up for about 3 months to confirm the diagnosis of SZ. All participants were assessed for SZ by two psychiatrists according to the Structured Clinical Interview for DSM-IV. The criteria for selecting patients included: (1) age between 18 and 55 years old; (2) acute onset at admission; (3) course of disease ≤ 2 years; (4) never taking antipsychotic drugs; (5) no physical disease. Patients with a personal history of alcohol or substance dependence (except for smoking) were excluded.

A total of 232 age- and sex-matched HC were also recruited from the communities in Beijing. Through the questionnaire survey, the researcher obtained demographic data, lifestyle (such as smoking history, drug abuse) and physical conditions. Those with a personal history of mental illness, alcohol or substance dependence (except for smoking) were excluded.

The study was approved by the Ethical Board of Beijing Hui-Long-Guan hospital (SCH-A01), and all participants filled out and signed a consent form after understanding the detailed research plan.

### 2.2. Clinical Symptom and Cognitive Function Measurements

Two experienced psychiatrists used PANSS to assess patients’ psychiatric symptoms [43]. These two psychiatrists attended a training curriculum on the use of PANSS. After the course, the inter-rater reliability was examined by inter-rater correlation coefficient (ICC) of the total PANSS score, which was 0.82. RBANS was applied to assess cognitive function of participants [44]. The Chinese version of RBANS has been translated by our research team, and we have also examined its test reliability in HC and SZ patients [45]. RBANS contains 12 subtests, which can be converted into five subscales (immediate memory, visuospatial/constructional, language, attention, delayed memory), and a total score.

### 2.3. Evaluation of MnSOD Activity

The researchers collected blood from the forearm veins of all participants from 7 a.m. to 9 a.m. after fasting the night before, and then coded each sample. An assistant, who was blind for clinical condition of all participants measured MnSOD activity according to established procedures [46,47]. Briefly, the activity of MnSOD in plasma was recorded in units per milliliter of plasma, and was measured by the cyanide inhibition method, which was derived from the nitrite method [48]. The coefficients of variation of *MnSOD* were 8% (inter-assay) and 6% (intra-assay).

### 2.4. Genotyping

DNA was extracted according to the routine procedures. The *MnSOD* Ala-9Val polymorphism genotypes were determined as previously reported [23,49]. The following primers were used: 5′-ACGTTGGATGCTGTGCTTTCTCGTCTTCAG-3′ and 5′-ACGTTGGATGTTCTGCCTGGAGCCCAGATA-3′. An assistant performed genotyping tests twice (20%) to ensure accuracy. All concordance rates were at least 91.3%, and call rates were higher than 93.3%.

### 2.5. Statistical Analysis

Deviations of Hardy-Weinberg equilibrium (HWE) were evaluated by χ^2^ test of goodness of fit. *MnSOD* Ala-9Val allele and genotype frequencies were calculated for SZ patients and HC using chi-square test. Chi-square test and Analysis of Variance (ANOVA) were used to compare categorical variables and continuous variables, respectively. Pearson’s product-moment correlation was used to examine the correlation between variables. Since there was no homozygous variant Ala/Ala genotype in patients, and it only occurred in 0.9% of HC, the Ala/Ala genotype and Val/Ala genotype were combined into one group in the analysis. In addition, we used *MnSOD* genotype (Val/Val vs. Ala/Ala + Val/Ala) and diagnosis (patients vs. HC) as between-group factors, and used RBANS total and subscale scores or MnSOD activity as dependent variables, with sex, education, age, BMI and smoking status as covariates. The main effects of genotype, diagnosis and genotype × diagnosis interaction was examined. In addition, we modified multiple tests by using Bonferroni corrections. Finally, we applied stepwise regression analysis to assess whether *MnSOD* genotype could regulate the relation between MnSOD activity and cognitive performance. The RBANS total and its subscale scores were used as the dependent variables, and MnSOD activity was used as the independent variable in each *MnSOD* genotype group. Age, sex, education, smoking status, BMI, and age at first onset were used as covariates in the above-mentioned stepwise forward entry models. We adopted SPSS 22 version for data analysis. The significance degree of all *p* values was set to less than 0.05 (with two tails).

## 3. Results

The general and clinical characteristics of the patients and HC by *MnSOD* Ala-9Val genotype subgroup are shown in Table 1. No differences were found in age, sex and smoking status between genotype groups in patients, HC, or in the entire sample. Compared with the HC, SZ patients had a lower BMI (*p* < 0.001). Further, we found that there were significant differences in education level (years) between genotypes in the whole sample or patients (both *p* < 0.05), indicating that the education level of Ala carriers was significantly higher than that of Val homozygotes. In addition, in SZ patients no differences were found in the PANSS total score and its subscale scores or the age of onset between different Ala-9Val polymorphism.

The *MnSOD* allele and genotype frequencies are shown in Table 2. The genotype distributions of *MnSOD* gene in patients and HC were consistent with HWE (both *p* > 0.05). No significant difference was found in the *MnSOD* genotype and allele distribution between patients and healthy individuals. In addition, there were no significant gender differences in *MnSOD* allele distribution and genotype among all participants or when healthy participants and patients were analyzed separately (all *p* > 0.05).

### 3.1. Genotype Effects on Performance of Cognition between Patients and HC

Table 3 summarizes the RBANS total and RBANS subscale scores grouped by *MnSOD* Ala-9Val polymorphism. As expected, we found that patients had lower total RBANS scores and lower scores on all subscales than HC, except for the visuospatial/constructional index (all *p* < 0.001). After adding sex, age, education, BMI and smoking as covariates, these differences were still significant (all *p* < 0.01).

According to Table 3, there was no genotype × diagnosis interaction on the RBANS total score or any index scores. We also did not observe significant differences in RBANS total or any index scores between the genotype subgroups of the combined group or when HC and SZ patients and were examined separately (all *p* > 0.05).

### 3.2. Effect of Ala-9Val Polymorphism on MnSOD Activity between Patients and Healthy Participants

The activity of MnSOD was analyzed in 215 UMFE SZ patients and 205 HC. Showing in the Table 3, the activity of plasma MnSOD in SZ patients was higher than that of HC (24.3 ± 15.3 U/mL vs. 22.0 ± 13.5 U/mL, F _(1,416)_ = 8.51, *p* < 0.01). After controlling for age, sex, education level, smoking status and BMI, the difference remained significant (F _(6,375)_ = 4.63, *p* < 0.05). In addition, there was a genotype × diagnosis interaction on MnSOD activity (F _(1,416)_ = 9.15, *p* = 0.003), which was still significant after Bonferroni correction (Table 3).

Patients with Ala alleles had higher MnSOD activity than patients with Val homozygote (F _(1,213)_ = 8.46, *p* = 0.004; Bonferroni corrected *p* < 0.05). After controlling for age, sex, education level, BMI and smoking status, there was still a significant difference in MnSOD activity between patients with Ala alleles and those with Val homozygote (F _(5,180)_ = 11.35, *p* < 0.01, Bonferroni corrected, *p* < 0.05). However, there was no difference in MnSOD activity between *MnSOD* genotypes in the HC (F _(1,203)_ = 1.80, *p* = 0.18).

### 3.3. MnSOD Activity and Performance of Cognition: Associations with MnSOD Ala-9Val Polymorphism

Pearson correlation analysis exhibited that only in patients, MnSOD activity was inversely correlated with immediate memory (r = −0.15, df = 209, *p* = 0.027), visuospatial/constructional (r = −0.17, df = 209, *p* = 0.012), attention (r = −0.19, df = 209, *p* < 0.01), and RBANS total scores (r = −0.19, df = 209, *p* < 0.01). Further, using gender and age as covariates, partial correlation analysis showed that these correlations remained significant (all *p* < 0.05). However, only the *p* value between MnSOD activity and attention score or RBANS total score passed Bonferroni correction (both corrected *p* < 0.05).

**Figure 1:**
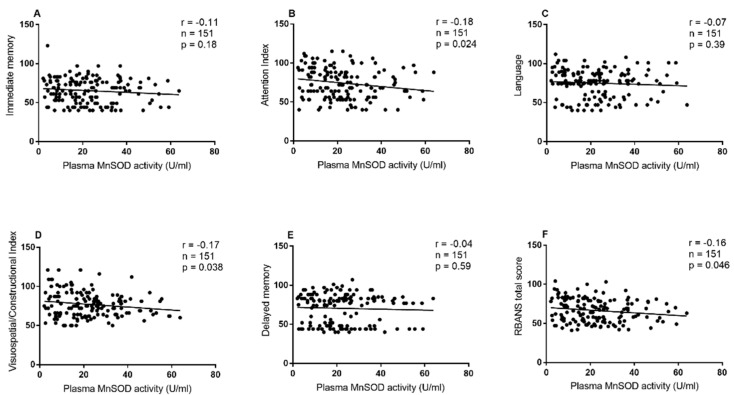
Association between MnSOD activity and cognitive performance in schizophrenia patients with the Val homozygote. Association between MnSOD activity and (**A**) Immediate memory, (**B**) Attention index, (**C**) Language, (**D**) Visuospatial/Constructional Index, (**E**) Delayed memory and (**F**) RBANS total score in schizophrenia patients with the Val homozygote.

As shown in Table 4 and Figure 1, in patients with Val homozygote, MnSOD activity was remarkably correlated with RBANS total score (r = −0.16, df = 151, *p* = 0.046), attention (r = −0.18, df = 151, *p* = 0.024), or visuospatial/constructional index (r = −0.17, df = 151, *p* = 0.038). Further, partial correlation analysis showed that these correlations remain significant when gender and age were used as covariates (all *p* < 0.05). However, there was no significant association between MnSOD activity and RBANSS scores in Ala allele carriers. Further regression analysis showed that MnSOD activity was significantly correlated with RBANS total score (beta = −0.17, t = −0.38, *p* = 0.04), attention index (beta = −0.23, t = −2.01, *p* = 0.045) and visuospatial/constructional index (beta = −0.22, t = −2.39, *p* = 0.018).

In the HC, there was no significant correlation between MnSOD activity and RBANS scores in the whole group or in the *MnSOD* genotype subgroups (all *p* > 0.05).

## 4. Discussion

Our study had several main results. (1) *MnSOD* Ala-9Val polymorphism may not directly contribute to SZ susceptibility. (2) Cognitive impairment was not dependent on *MnSOD* polymorphism in SZ patients. (3) Ala variant was associated with higher MnSOD activity in SZ patients, but not in HC. (4) Only in SZ patients with Val homozygote, MnSOD activity was remarkably associated with cognitive performance.

### 4.1. Effects of Val-9Ala Polymorphism on the Susceptibility of SZ

Previous studies on *MnSOD* Ala-9Val polymorphism and SZ susceptibility are inconsistent [23,24,25,26,27]. For example, some studies have found that *MnSOD* Ala-9Val genotype may be concerned with the physiological pathogenesis of SZ [24,25]. However, several other studies have not revealed the association between Ala-9Val polymorphism and SZ [15,23,26,27]. Different ethnic backgrounds may be the main reason for the inconsistency of these results. It is noteworthy that compared with Caucasians and African Americans, most Asians have higher frequencies of Val/Val genotypes and Val alleles. It is reported that the frequency of Ala alleles in Asians ranges from 9.4% to 16.3% [27,50,51], while the frequency in Caucasians is around 50% [26,52]. In this study, the allele frequency in the healthy group was 12.9%, which was similar to the results of other studies among Asian populations. In addition to ethnic differences in the *MnSOD* Ala-9Val polymorphism, the general heterogeneity of SZ diagnosis, small gene effect and population stratification may be important factors that lead to inconsistent results.

### 4.2. MnSOD Ala-9Val Polymorphism on Cognitive Function in Participants with SZ

Patients with UMFE SZ showed significant cognitive decline in the RBANS total score and four subscales including immediate memory, attention, language and delayed memory, which replicate our previous results in first-episode [53,54,55] and chronic SZ patients [56,57]. These consistent results indicate that cognitive impairment appears in the early stages s of the disease and seems to be an essential feature of SZ patients.

In this study, there were no significant differences in all RBANS scores in the three genotypes of the patient and healthy groups, suggesting that the *MnSOD* genotype may have no significant regulatory effect on the cognitive dimensions we measured. Our study is the first to systematically focus on the association between *MnSOD* variant and cognitive function in patients with UMFE SZ. This result is inconsistent with our previous study, which showed that the *MnSOD* Val-9Ala genotype was associated with the attention domain of patients with chronic SZ [23]. This inconsistent result may be due to the fact that the average age of first-episode patients is younger than that of chronic SZ patients (approximately 27 years vs. 48 years). Based on the resource regulation hypothesis, with the increase of age, the gradual shortage of brain resources leads to enhanced regulation of genetic variation on cognition, brain structure and function, so genetic variation plays a more important role in the aging process [58,59].

There is growing evidence showing that compared with the elderly, the genetic effects of young people are moderate or non-existent [60]. Previous studies have shown that the functional *MnSOD* variants are not an independent risk factor for amnestic mild cognitive impairment, which is similar to our results [61]. Further studies are needed to recruit relatively older samples to determine whether there is an age-dependent relationship between *MnSOD* variants and cognitive performance.

### 4.3. Increased MnSOD Level and Dysfunction of Cognition: Relationship to MnSOD Val-9Ala Genotype

We found that compared with HC, UMFE SZ patients had higher MnSOD activity, which was in line with our previous research results [17] and other research results [18,51]. However, some studies have found that there is no change or decrease in MnSOD activity in patients with first episode [62] or chronic SZ [15,16,23]. A variety of factors may cause these inconsistent results, including but not limited to disease state (chronic or acute onset), history of antipsychotic drugs, test sources (serum, plasma, platelet or brain tissue), obesity and *MnSOD* polymorphism [63,64]. In addition, it has been found that 8-hydroxy-2 deoxyguanosine, a common biomarker in DNA oxidative damage, has increased in patients with first-episode SZ [65]. Therefore, we speculate that the increased MnSOD activity is a compensatory response to excessive detoxification of ROS.

Furthermore, our study found that the MnSOD activity in plasma was regulated by *MnSOD* genotype, indicating that the MnSOD activity of Ala carriers was higher than that of Val homozygous patients, but there was no such effect in the HC. As far as we know, this study is one of the first to reveal that plasma MnSOD activity is regulated by *MnSOD* genotype in patients with UMFE SZ. This result is different from our previous finding that *MnSOD* genotype has no effect on MnSOD activity in chronic SZ patients [23]. Several factors, such as antipsychotic treatment, course of disease, and disease status (acute or chronic) may lead to this inconsistent result. Several authors have found similar results in other diseases, such as diabetes mellitus and prostate cancer, suggesting that the -9Ala allele may be associated with higher MnSOD activity, which is similar to previous results [64,66]. Ala-9Val as a functional polymorphism of the *MnSOD* gene, its variant from C to T represents the amino acid change from Val to Ala, resulting in the chromosome structure alteration in the targeted domain from the beta-sheet to the alpha-helix. Compared with the beta-sheet structure, the alpha-helical structure containing the Ala precursor enhances the transport efficiency of the enzyme to the mitochondria matrix and increases the antioxidant defense enzyme against ROS [22]. In addition, our study did not detect the effect of Ala-9Val polymorphism on SZ susceptibility. Considering the influence of Ala alleles on MnSOD activity, it is speculated that Ala-9Val mutants may affect the OS process involved in MnSOD, thereby participating in the development of SZ.

We also found that increased MnSOD activity was inversely related to general cognitive performance and several cognitive domains, especially with the poor attention index in UMFE SZ patients, but not in HC. This result is similar to our previous studies in the first-episode [62] and chronic SZ patients [16,37]. Another study found that total antioxidant capacity (TAC) was particularly associated with attention index in non-affective SZ patients [67]. Furthermore, a previous study also found that in patients with recurrent depressive disorder, the MnSOD expression was significantly negatively correlated with some cognitive performance tests including Trial Making test and Stroop test [41]. These studies indicate that OS may play an essential role in cognitive impairment of patients with mental illness. However, the negative correlation between MnSOD activity and cognition exceeded our expectation. It should be pointed out that OS is caused by oxidative damage due to the imbalance between pro-oxidants and antioxidants, so high levels of antioxidant enzymes may not indicate low OS damage [68]. A previous study demonstrated that the activity of antioxidant enzymes was found enhanced in the elderly, while indicators representing OS damage, including 8-hydroxy-2′-deoxyguanosine and protein carbonyls, were also increased [69]. Based on these findings, we suppose that the increased MnSOD is a compensatory response to OS to protect the human body from free radical damage. The cognitive impairment of SZ patients may be partly attributed to the damage of OS. As an antioxidant enzyme that scavenges ROS in mitochondria, MnSOD participates in neurocognitive processes mainly through the interaction of antioxidant enzymes, increased OS and cellular destruction [70,71].

It has been found that in neurodegenerative diseases such as AD, the expression of MnSOD in hippocampal neurons is downregulated, while overexpression of MnSOD reduces hippocampal superoxide and Aβ plaques, and prevents learning and memory deficits associated with AD [33,72]. At present, it is not clear how MnSOD activity affects cognitive function, but when the balance of ROS and antioxidant enzymes is disrupted, it will hinder the formation of LTP, the molecular basis of learning [73,74]. In addition, ROS is also an important signaling molecule, which is necessary for synaptic plasticity and construction of memory [75].

Another impressive finding of this study was that *MnSOD* polymorphism regulated the relationship between cognitive performance and MnSOD activity in UMFE SZ patients, that is, a remarkable inverse relationship between MnSOD activity and attention, visuospatial/constructure index or RBANS total scores was observed only in patients with Val homozygote, but there was no such correlation in Ala carriers. The difference in plasma MnSOD-cognitive function between different *MnSOD* genotypes in UMFE SZ patients gives a hint about the unexpected discovery of higher MnSOD activity with more damage of cognitive performance. It was known that the *MnSOD* gene-9Ala variant, but not the -9Val allele, has an alpha-helical structure. This alpha-helical structure helps to transport the MnSOD precursor protein into the mitochondria more effectively, that is, individuals with *MnSOD* -9Ala genotypes can transfer more MnSOD precursor to mitochondria than individuals with-9Val genotypes, thus eliminating more excessive detoxification of ROS [22]. As a result, only patients with Ala carrier produced additional MnSOD, while patients with Val/Val did not produce more MnSOD to protect against the cognitive impairment caused by ROS-induced neuronal damage. Interestingly, we did find that MnSOD activity in Val homozygous patients was lower than that in Ala carriers, suggesting that patients with Val homozygotes may suffer more severe ROS damage. Therefore, in UMFE SZ patients with Val homozygote, when ROS production increases, leading to cognitive impairment, it induces more MnSOD production as a compensatory increase, thus showing a negative interrelationship between MnSOD activity and cognitive performance. Certainly, these are our speculations, and further longitudinal studies are needed in SZ patients to clarify the relationship between peripheral blood MnSOD activity and cognitive impairment, and to further investigate how *MnSOD* genotype affects the relationship between cognitive deficits and MnSOD activity.

Some limitations of this research are as follows. First, we examined the activity of only one antioxidant enzyme, which is involved in only one part of the action of the antioxidant defense system. Both antioxidant enzymes and non-enzymes should be included in the measurement to have a more detailed knowledge about the antioxidant defense system in future studies. Second, in this study, we did not measure the protein expression of Mn-SOD. Therefore, we cannot directly demonstrate that the regulation of the activity is due to the presence of the *Mn-SOD* gene polymorphism. Furthermore, as no other parameters of oxidative stress were measured, it is possible that the change in Mn-SOD activity is due to another imbalance in the redox system. Third, specifically, the higher activity of MnSOD in plasma would be expected to produce higher levels of hydrogen peroxide, H_2_O_2_, which could be directly measured with appropriate dyes. Moreover, the excess H_2_O_2_ would be expected to lead to elevated markers of oxidative damage to proteins and lipids, for example protein carbonyls and protein-bound 4-hydroxynonenal (HNE) or isoprostanes, but definitely not thiobarbituric acid reactive substance (TBARS). In this study, unfortunately, we did not measure H_2_O_2_ levels, which should be remedied in the future studies to explore whether our results in this study are consistent with the expectations of elevated H_2_O_2_. Fourth, this study only took Han Chinese as the research subjects. Since there are significant differences in Ala allele frequencies between Asians and whites, our findings need to be verified in different races in future studies. Fifth, we only detected one polymorphism of *MnSOD* gene in this study. Previous studies have shown that other polymorphisms (such as Ile58Thr) also regulate the expression of *MnSOD* gene, which should be included in future studies to fully understand the effects of *MnSOD* gene mutations [76]. Sixth, due to the cross-sectional design of this study, it is largely observational in nature rather than, and we could not draw a causal relationship between MnSOD and cognitive performance in SZ patients, which may be clarified in a future study with a longitudinal design.

In summary, this study provides evidence for the correlation between *MnSOD* polymorphism, MnSOD activity and cognitive deficits in patients with UMFE SZ. Our study showed that patients with UMFE SZ had extensive cognitive deficits and higher MnSOD activity. Moreover, our results showed that MnSOD activity was negatively associated with general cognitive function, especially attention in SZ patients, but not in HC. More importantly, the relationship between cognitive performance and MnSOD activity was regulated by *MnSOD* Ala-9Vla polymorphism in these SZ patients. These results indicate that in the early stages of the disease, the peripheral MnSOD activity and Ala-9Vla gene polymorphism may be involved in the pathological mechanism of cognitive dysfunction in SZ patients. Among our findings, the important result was that MnSOD activity and cognitive performance were significantly correlated in SZ patients with Val homozygotes, suggesting that oxidative stress may be implicated in the psychopathological mechanisms of cognitive impairment of schizophrenia patients, and the use of antioxidants is a possible option for the treatment of cognitive decline in patients with first-episode SZ in the clinical practice. In view of the difference in Ala allele frequency between Asians and whites, our finding that the *MnSOD* genotypes regulated the association between MnSOD activity and cognitive impairment may only be specific for Chinese samples. Future studies need to include people from different ethnic backgrounds to verify the findings of this study.

## Figures and Tables

**Table 1:** Demographics and clinical characteristics in first-episode patients and healthy controls grouped by *MnSOD* Val-9Ala genotype.

	Schizophrenia (*n* = 234)					Healthy Controls (*n* = 232)				
Variable	Total	Ala/Ala +Ala/Val (*n* = 63)	Val/Val (*n* = 171)	χ2 or F	*p*	Total	Ala/Ala +Ala/Val (*n* = 58)	Val/Val (*n* = 174)	χ2 or F	*p*
Male (%)	137 (58.5%)	36 (57.1%)	101 (59.4%)	0.10	0.77	123 (53%)	31 (53.4%)	92 (52.9%)	0.01	0.94
Age (years)	27.1 ± 9.4	27.2 ± 9.0	27.1 ± 9.6	0.01	0.91	28.7 ± 11.0	28.5 ± 9.9	28.8 ± 11.3	0.04	0.85
Education (years)	9.2 ± 3.9	10.2 ± 3.9 *	8.9 ± 3.8	5.52	**0.02**	9.6 ± 3.1	10.0 ± 3.0	9.4 ± 3.2	1.21	0.27
BMI (kg/m^2^)	21.4 ± 3.4	22.0 ± 3.4	21.2 ± 3.4	2.1	0.15	24.4 ± 3.8	24.4 ± 3.9	24.4 ± 3.7	0.01	0.96
Smokers (%)	60 (25.6%)	17 (28.8%)	43 (25.7%)	0.21	0.73	74 (31.9%)	17 (29.8%)	57 (33.1%)	0.22	0.74
Age of onset (year)	26.0 ± 9.5	26.2 ± 8.9	25.9 ± 9.7	0.05	0.82					
PANSS total score	75.5 ± 16.7	74.9 ± 16.5	75.7 ± 16.8	0.11	0.74					
*p* subscore	21.4 ± 6.3	21.6 ± 5.9	21.3 ± 6.4	0.11	0.74					
N subscore	18.9 ± 7.0	18.4 ± 7.3	19.0 ± 6.9	0.37	0.55					
G subscore	35.3 ± 9.6	34.9 ± 8.9	35.4 ± 9.8	0.12	0.73					

Note: * refers to the comparison of genotype in the corresponding group.

**Table 2:** *MnSOD* allele and genotype distributions in patients with schizophrenia and healthy controls.

	Schizophrenia (*n* = 234)	Controls (*n* = 232)	F (*p*) Value
Allele frequency (%)			0.06 (0.81)
Ala	63 (13.5%)	60 (12.9%)	
Val	405 (86.5%)	404 (87.1%)	
Genotype frequency (%)			2.43 (0.30)
Ala/Ala	0 (0%)	2 (0.9%)	
Ala/Val	63 (26.9%)	56 (24.1%)	
Val/Val	171 (73.1%)	174 (75%)	

**Table 3:** Cognitive performance and plasma MnSOD activity in first-episode schizophrenia patients and normal controls grouped by *MnSOD* Ala-9Val genotype.

	Schizophrenia (*n* = 228)		Healthy Control (*n* = 228)		F _case vs. control_ (*p* Value)	F _genotype_ (*p* Value)	F _interaction_ (*p* Value)
Cognitive Index	Ala/Ala + Ala/Val (*n* = 63)	Val/Val (*n* = 165)	Ala/Ala + Ala/Val (*n* = 58)	Val/Val (*n* = 170)			
Immediate memory	66.2 ± 18.5	64.8 ± 16.0	74.8 ± 13.7	75.5 ± 17.7	**29.7 (<0.001)**	0.05 (0.82)	0.34 (0.56)
Attention	73.7 ± 22.3	74.7 ± 19.4	88.5 ± 19.1	87.6 ± 19.1	**44.2 (<0.001)**	0.01 (0.98)	0.21(0.65)
Language	76.5 ± 20.0	75.4 ± 18.1	92.6 ± 13.2	94.8 ± 11.9	**112.8 (<0.001)**	0.08 (0.78)	0.98(0.32)
Visuospatial/constructional	78.3 ± 18.9	77.0 ± 16.2	79.6 ± 14.4	78.6 ± 15.1	0.73 (0.39)	0.49 (0.49)	0.01 (0.95)
Delayed memory	70.5 ± 21.4	69.4 ± 19.6	83.0 ± 13.4	86.8 ± 14.2	**66.0 (<0.001)**	0.53 (0.47)	1.73 (0.19)
Total	67.6 ± 19.0	66.2 ± 14.8	78.6 ± 12.6	80.2 ± 14.2	**61.5 (<0.001)**	0.01 (0.95)	0.90 (0.34)
MnSOD (U/mL)	29.2 ± 18.0	22.5 ± 13.8	19.8 ± 11.2	22.7 ± 14.1	**8.51 (<0.01)**	1.44 (0.23)	**9.15 (<0.01)**

**Table 4:** Relationships between MnSOD activity and cognitive domain scores across *MnSOD* genotype in schizophrenia patients.

	Ala Carriers (*n* = 58)		Val Homozygotes (*n* = 151)	
Cognitive Domains	Pearson r	*p*	Pearson r	*p*
Immediate memory	−0.23	0.08	−0.11	0.18
Attention	−0.20	0.14	−0.18	**0.024**
Language	−0.18	0.18	−0.07	0.39
Visuospatial/Constructional	−0.18	0.17	−0.17	**0.038**
Delayed memory	−0.11	0.42	−0.04	0.59
Total	−0.24	0.08	−0.16	**0.046**

## Data Availability

The data presented in this study are available on request from the corresponding author.
